# Custom Design of a Humidifier Chamber for *InMeso* Crystallization

**DOI:** 10.1021/acs.cgd.3c01034

**Published:** 2023-12-12

**Authors:** Egor Marin, Kirill Kovalev, Therese Poelman, Rick Veenstra, Valentin Borshchevskiy, Albert Guskov

**Affiliations:** † Groningen Institute for Biomolecular Sciences and Biotechnology, University of Groningen, 9747AG Groningen, The Netherlands; ‡ European Molecular Biology Laboratory, EMBL Hamburg c/o DESY, 22607 Hamburg, Germany; § Forschungszentrum Jülich GmbH, Leo-Brandt-Straße, 52428 Jülich, Germany; ⊥ University of Groningen, 9747AG Groningen, The Netherlands

## Abstract

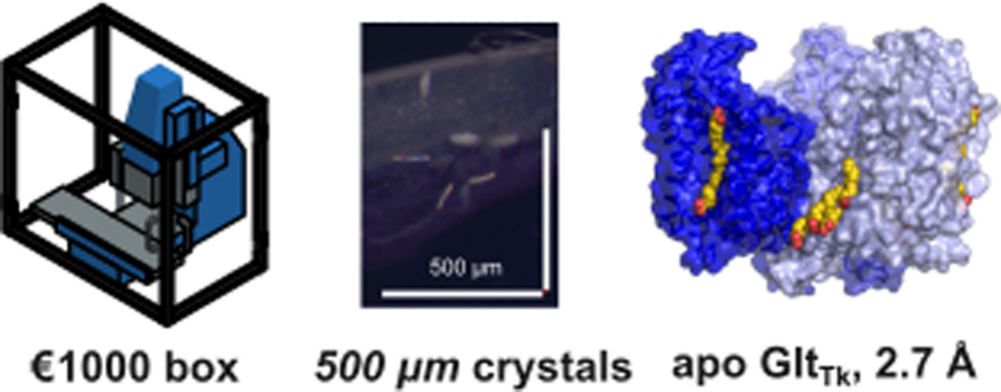

Membrane proteins are indispensable for every living organism, yet their structural organization remains underexplored. Despite the recent advancements in single-particle cryogenic electron microscopy and cryogenic electron tomography, which have significantly increased the structural coverage of membrane proteins across various kingdoms, certain scientific methods, such as time-resolved crystallography, still mostly rely on crystallization techniques, such as lipidic cubic phase (LCP) or *in meso* crystallization. In this study, we present an open-access blueprint for a humidity control chamber designed for LCP/*in meso* crystallization experiments using a Gryphon crystallization robot. Using this chamber, we have obtained crystals of a transmembrane aspartate transporter Glt_Tk_ from *Thermococcus kodakarensis* in a lipidic environment using *in meso* crystallization. The data collected from these crystals allowed us to perform an analysis of lipids bound to the transporter. With this publication of our open-access design of a humidity chamber, we aim to improve the accessibility of *in meso* protein crystallization for the scientific community.

## Abstract 2

A custom-made humidifier chamber for the Gryphon crystallization robot increases the reproducibility and quality of crystals.

## Introduction

Membrane-embedded proteins play numerous essential roles in all kingdoms of life. Namely, transmembrane proteins, spanning across cell membranes, allow transfer of matter and information from one side of the membrane to another, otherwise severely hindered by the semipermeable nature of biological membranes.^1−5^

To understand the mechanisms of such transfer, the structural insights are essential; in most of the cases, the structures of membrane proteins are obtained with single-particle cryogenic electron microscopy (cryo-EM) or macromolecular X-ray crystallography. For the latter, obtaining well-diffracting crystals is a prerequisite. Unfortunately, most of the membrane proteins suffer upon their extraction from the membrane; hence, many efforts were spent to develop a technique where membrane proteins can be crystallized in the membrane-like environment. For this purpose lipidic cubic phase (LCP) and *in meso* crystallization (meaning that the crystallization takes place in a lipidic cubic or mesophase, which are considered liquid crystalline states and resemble the membrane bilayer) methods have been developed,^6,7^ allowing to grow well-diffracting crystals yielding high-resolution three-dimensional structures of membrane proteins in a near-native lipidic environment. Overall, LCP/*in meso* crystallization has been shown to be applicable for various types of proteins yielding structures of prokaryotic and eukaryotic proteins, homo- and heteromultimers, and α-helical and β-barrel folds.^7−9^

Despite difficulties in dispensing of the viscous lipidic phase, the sample consumption in LCP/*in meso* crystallization is comparable to liquid-based *in surfo* crystallization methods (where a protein of interest is generally extracted from the membrane bilayer by solubilization with detergents); typically, tenths of milligrams of protein are used for a single plate. Such low consumption is achieved via accurate dispensing of nanoliter-sized droplets of lipid phase (50–150 nL) onto a glass plate, which is subsequently covered with the precipitant.^10^

Due to the small size of crystallization droplets, crystallization success and reproducibility require precise control over the environment’s humidity. In some crystallization robots, such as NT8 (Formulatrix), this is achieved by placing the crystallization setup into a built-in humidity chamber. In this chamber, the humidity is controlled and is usually kept around 100% during the dispensing process. However, in some other crystallization robots, e.g., in Gryphon (Art Robbins Instruments), no such chamber is present. Moreover, for this robot, all lipid droplets are covered with precipitant only after their dispersion into all 96 wells. This significantly increases the “dry” time for the lipid droplets, which can cause undesirable effects, although successful LCP crystallizations have also been reported.^11−13^

In this work, we present an open-access blueprint for the humidity chamber for the Gryphon crystallization robot to overcome the aforementioned shortcomings. This chamber does not require any commercial parts and can be easily produced in a standard workshop. In the proposed setup, the humidity inside the chamber can be increased with any commercially available vaporizer and controlled with a humidity sensor.

To test our setup, we performed *in meso* crystallization trials with a glutamate transporter homologue Glt_Tk_,^14−16^ which is widely used as a study model for investigations on solute carrier family 1 (SLC1) of transporters alongside another archaeal homologue Glt_Ph_.^17−19^ Structurally, Glt_Tk_ is a homotrimer, with every monomer consisting of a rigid scaffold domain that also serves as an oligomerization interface and an elevator domain, that undergoes conformational change during substrate transport. The transport domain of Glt_Tk_ binds three sodium ions and a substrate aspartate molecule, occluding it by two helical hairpin elements, HP1 and HP2.^20^ The latter hairpin is known to serve as both an intra- and extracellular gate. Domains have been shown to move independently between monomers in both Glt_Tk_ and its close homologue Glt_Ph_.^17^ Multiple structural snapshots of Glt_Tk_ transport states have been obtained based on the conformation of each monomer: 3out, 2out:1in, 1out:2in, and intermediate states responsible for ligand binding, such as intermediate outward-occluded and outward-open.^16^

Prior to this study, multiple structures of Glt_Tk_ have been obtained using either *in surfo* crystallization or cryo-EM techniques. For the former these include substrate-free (*apo*) structures and structures in complex with l- and d-aspartate, and photocaged and photoswitchable compounds have been reported,^21−24^ notably all in the outward conformation. For the latter approach different combinations of outward and inward conformations have been obtained (presumably due to relief of crystal symmetry constraint): 2in:1out, 1in:2out, and 3out in the presence of the substrate, as well as a sodium-only structure (in intermediate outward-occluded state), a structure in complex with a TBOA inhibitor (in outward-open conformation^16^), and a structure with a synthetic nanobody bound to an outward-facing transport domain.^15^

Importantly all our previous attempts of LCP/*in meso* Glt_Tk_ crystallization using the Gryphon robot were unsuccessful; however, with the designed chamber we, for the first time, obtained Glt_Tk_ crystals grown in the native-membrane-mimetic phase. These nonoptimized “hit” crystals allowed us to solve two crystal structures belonging to two different space groups at resolutions of 2.7 and 3.2 Å. The quality of these data is comparable to previously published Glt_Tk_ crystallographic data obtained *in surfo*, although the latter one required a time-consuming optimization process to achieve the same level of diffraction resolution. Analysis of these structures revealed an accessible hydrophobic cavity which is occupied by a lipid molecule. To investigate whether this density is observed in different conformational states, we have re-evaluated previously obtained cryo-EM data^16^ which revealed a density patch at the same location, present in both outward and inward conformations of the transporter. The lipid molecule at this position can have an important implication for a protein’s function and/or stability.

Given the obtained results, we believe that our open-access chamber design will improve reproducibility and success rates for the LCP/*in meso* crystallization experiments using Gryphon robots.

## Materials and Methods

### Protein Expression and Purification

The protein was purified as described previously.^14^ Briefly, C-terminally 8-His-tagged Glt_Tk_ in a pBAD24-derived plasmid was expressed in *Escherichia coli* MC1061. Cells were grown in LB medium with 100 μg/mL ampicillin at 37 °C and 200 rpm and induced with 0.05% l-arabinose for 3 h upon reaching 0.8 OD_600_. After the cells were harvested and broken, the resuspension buffer contained no sodium to ensure purification of the *apo* structure. Obtained membrane vesicles were solubilized in 50 mM Tris-HCl, pH 8.0, 300 mM KCl, and 1% *n*-dodecyl-β-d-maltoside (DDM) for 1 h at 4 °C. After ultracentrifugation (30 min, 265,000 g, 4 °C), the supernatant was incubated with Ni-sepharose resin in a gravity-flow column for 1 h at 4 °C. After that, the column was washed with 50 mM Tris-HCl, pH 8.0, 300 mM KCl, 0.15% *n*-decyl-β-d-maltoside (DM), and 60 mM imidazole, pH 8.0, and the protein was eluted with the same buffer containing 500 mM imidazole. The last purification step was performed on size exclusion chromatography column Superdex 200 10/300 (GE Healthcare) in 10 mM HEPES KOH, pH 8.0, 100 mM KCl, and 0.15% DM.

### *In Meso* Crystallization

**Figure 1:**
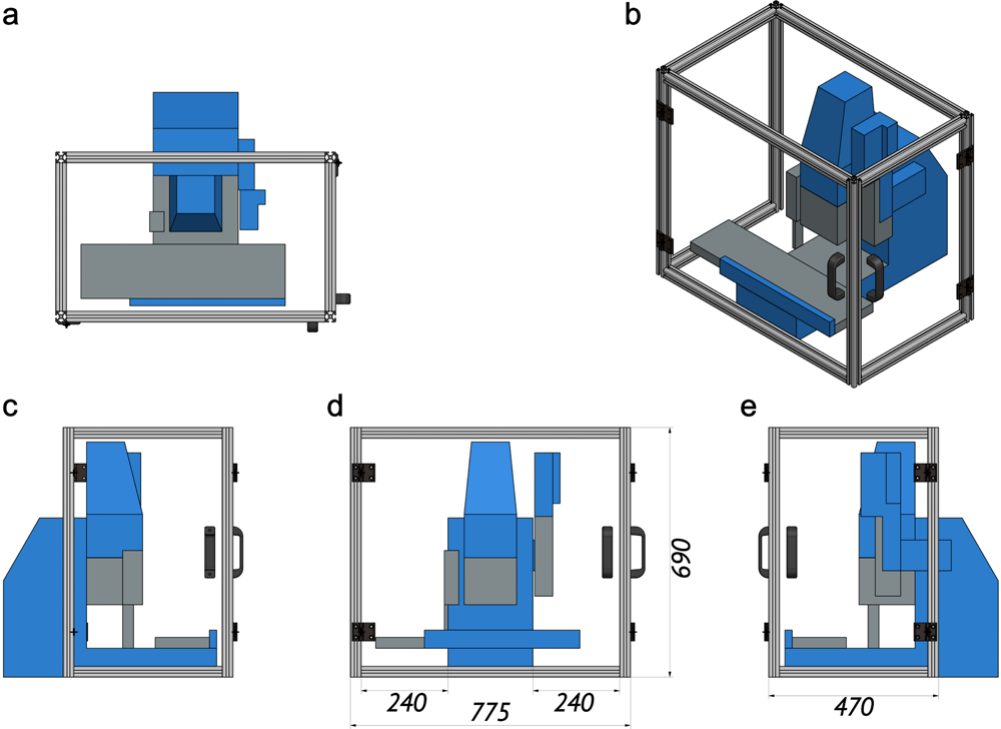
Schematics of the humidity chamber for the Gryphon crystallization robot in (a) top, (b) isometric, (c) left, (d) front, and (e) right views. Dimensions are shown in millimeters.

The crystals of Glt_Tk_ were grown with an *in meso* approach,^6^ similar to that used in our previous works^25,26^ but with a Gryphon crystallization robot (Art Robbins Instruments, ARI). In particular, the solubilized protein (20 mg/mL) in the gel-filtration buffer was mixed with monoolein and melted at 42 °C (MO, Nu-Chek Prep) in a 3:2 ratio (lipid:protein) to form a lipidic mesophase. The mesophase was homogenized in coupled syringes (Hamilton and Art Robbins Instruments, ARI) by transferring the mesophase from one syringe to another until a homogeneous gel-like material was formed. The mesophase was then transferred to the ARI syringe for further use with the Gryphon crystallization robot embedded in the humidity box (see Figure 1 and Supplementary Figure 1).

Prior to crystallization, the humidity chamber was saturated with the water vapor using a humidifier device at maximum power as a vapor source and a humidity sensor as the control. Saturation >90% was reached within 10 min, at the temperature of 20 °C and room humidity around 40%. After that, the humidifier power was reduced to 50% during the crystallization process to prevent excessive formation of water droplets on available surfaces, such as the crystallization plate. After every time the chamber was opened to insert a syringe or change the crystallization screen or plate, the humidity inside the chamber was brought back to >90% within a couple of minutes before the drop dispensing began.

For the crystallization, 150 nL drops of a protein–mesophase mixture were spotted on a 96-well LCP glass sandwich plate (Marienfeld) and overlaid with 400 nL of precipitant solution by the Gryphon crystallization robot inside the humidity chamber (Figure 1). For all crystallization screenings we used the CubicPhase 1 crystallization kit (Qiagen). The protein crystals were obtained in two space groups. The best-looking crystals in the *H*3_2_ space group were obtained in 1.6 M ammonium phosphate, pH 5.0, as a precipitant. The best-looking crystals in the *P*6_3_22 space group were obtained in 1 M ammonium sulfate and 100 mM sodium acetate, pH 4.9, as a precipitant. The crystals were grown at 21 °C and reached their full size in 1 month (Supplementary Figure 2).

### Collection of Crystallographic Data

Once the crystals reached their final size after 1 month, crystallization wells were opened and drops containing the protein–mesophase mixture were covered with 100 μL of the precipitant solution. Crystals were harvested with MicroMount loops (MiTiGen) and flash-frozen in liquid nitrogen for subsequent data collection. Data were collected at the Petra III synchrotron, beamline P13 (Hamburg, Germany).

### Structure Solution and Refinement

Data were integrated using XDS and scaled with XSCALE.^27,28^ Molecular replacement was performed with phenix.phaser^29^ using the Glt_Tk_*apo* model (PDB ID 5DWY) as a template. After a few rounds of refinement in phenix^30,31^ and manual rebuilding in Coot,^32^ the paired refinement^33^ was performed to determine the final resolution cutoff for subsequent refinement. Data processing and refinement statistics are reported in Supplementary Table 1.^34^

### Single-Particle Cryo-EM Data Reprocessing

All steps of data reprocessing were performed using cryoSPARC v.4.0.2^35^ using the data sets described in Arkhipova et al.^16^ and are summarized in Supplementary Figure 4. Motion correction and CTF estimation were performed with default settings for all three data sets. Respective EMDB^36^ volumes (EMD-10633 as “2in:1out”, EMD-10634 as “1in:2out”, and EMD-10635 as “saturated”) were used for particle picking. For note, saturation here means the ratio of protein to l-aspartate; for the saturated condition it is 1:3, which brings all the transport domains in an outward (out) position, whereas for the unsaturated condition it is 3:1, which generates an ensemble of states where some transport domains are in an outward position and some are in inward (in) positions.

For the unsaturated data set, containing both 1in:2out and 2in:1out particles, the picked particles were extracted with 2x binning (256 to 128 px). An initial set of particles was cleaned using two rounds of 2D classification (first round, 80 classes, 80 iterations, batch size 400, use clamp-solvent = true; second round, 40 classes, 40 iterations, use clamp-solvent = true). After that, particles were cleaned using a “3D classification” (*ab initio* model generation with seven classes, followed by heterogeneous refinement), producing 81,031 and 87,270 particles for the nonbinned refinement for the 2out:1in and 1out:2in conformations, respectively. These particles were re-extracted with a 384 px box size, followed by nonuniform refinement (with per-particle CTF and defocus refinement) and local refinement with automatically generated mask, yielding final resolutions of 3.2 Å for both the 2out:1in and 1out:2in conformations–an almost 0.2 Å improvement compared to previously reported reconstructions.^16^

For the saturated data set, the picked particles were extracted with 2x binning (256 to 128 px). An initial set of particles was cleaned using two rounds of 2D classification (first round, 80 classes, 80 iterations, batch size 400, use clamp-solvent = true; second round, 40 classes, 40 iterations, use clamp-solvent = true). After that, particles were cleaned using a “3D classification” (*ab initio* model generation with three classes, followed by heterogeneous refinement), producing 89,189 particles for the nonbinned refinement. These particles were re-extracted with a 384 px box size, followed by nonuniform refinement (with per-particle CTF and defocus refinement), yielding a final resolution of 3.3 Å–an ∼0.1 Å improvement compared to previously reported reconstructions.^16^

Based on improved densities, we modeled lipid molecules in hydrophobic grooves between adjacent chains in the saturated and 2out:1in conformations. We used POPC molecules as the scaffold and removed the blurred choline group and parts of the lipid tails unresolved in the maps. Lipid modeling and refinement was performed in Coot.^32^

## Results and Discussion

### Overall Design of the Humidity Box

The humidity chamber design consists of aluminum profiles (Boikon) with transparent polycarbonate plates in between, with estimated production costs around €1,000. At the front, there is a single door with a height of 630 mm and width of 715 mm for easy access inside the box. On the right side, there is one extra door with a height of 630 mm and width of 420 mm to change the LCP syringe when necessary. For the cables of the Gryphon robot, there are cutouts at the back of the box. The assembled humidity chamber is shown in Figure 1 and Supplementary Figure 1. The blueprint of the chamber in “stp” format is available as Supporting Information for this paper.

To maintain a humid environment inside the chamber, we used a humidifier device (Bionaire BU1300), which we put inside the humidity chamber, but basically any other commercial evaporator will work. To monitor humidity, we used a commercially available humidity sensor TFA Dostmann (model number 30.3045.IT), which provided fast enough feedback to monitor small changes in humidity and readjust the humidifier power; however, this is in principal optional.

### Crystals Obtained Using the Humidity Box for the Gryphon Robot

We tested the crystallization setup using an archaeal homologue of human glutamate transporters, Glt_Tk_. The trials were performed *in meso* as described in the Materials and Methods section. The protein crystals were obtained in two space groups, *H*3_2_ and *P*6_3_22 (Supplementary Figure 2), with a maximum of 500 μm in both cases. The best crystals were harvested for data collection, as described in the Materials and Methods. Notably, previous crystallization trials with the same crystallization robot but without the humidity chamber did not yield any crystals.

### Overall Structure of *In Meso* Crystallized *apo*-Glt_Tk_

**Figure 2:**
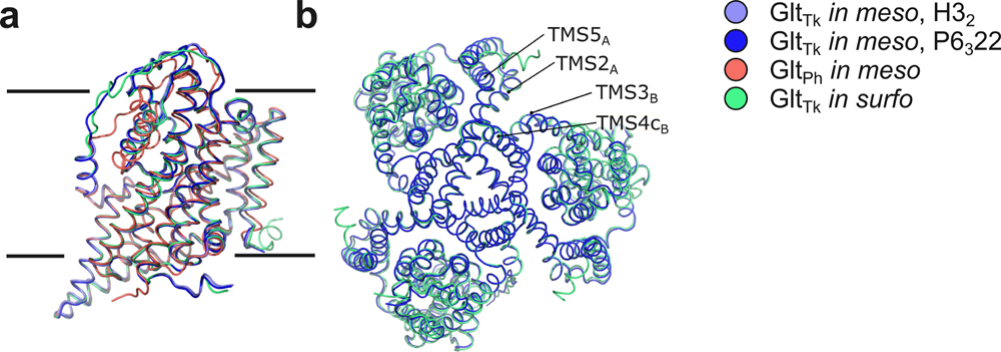
Superposition of the available Glt_Tk_ (5DWY, green) and Glt_Ph_ (7AHK, red) structures with the structures obtained in this study (*H*3_2_, light blue; *P*6_3_22, dark blue). (a) Side view of the monomer; (b) top view of the trimer.

Both crystal structures of Glt_Tk_ from crystals that appeared during initial screening *in meso* have a similar resolution as the Glt_Tk_ structures obtained by *in surfo* crystallization after extensive optimization.^22^ The obtained data allowed assignment of most of the residues, except for 119–124 amino acids in the 3–4 loop, as well as flexible C- and N-terminal residues. The crystal structures in the *H*3_2_ and *P*6_3_22 space groups are highly similar, with 0.3 Å α-helix C_α_-RMSD. Both crystal structures have one protomer of Glt_Tk_ in the asymmetric unit (AU) (see Supplementary Figure 3 for crystal packing), in contrast to all previously obtained Glt_Tk_ structures crystallized as a trimer in AU (Figure 2). A physiological trimer can be generated from three symmetry-related molecules, and the symmetry-completed trimer structure is very similar to the *in surfo* crystallized *apo*-Glt_Tk_ (PDB ID 5DWY) and *in meso* crystallized Glt_Ph_ (PDB ID 7AHK) structures with 1.0 Å α-helix C_α_-RMSD and 2.0 Å α-helix C_α_-RMSD, respectively.

### Lipids Placement

**Figure 3:**
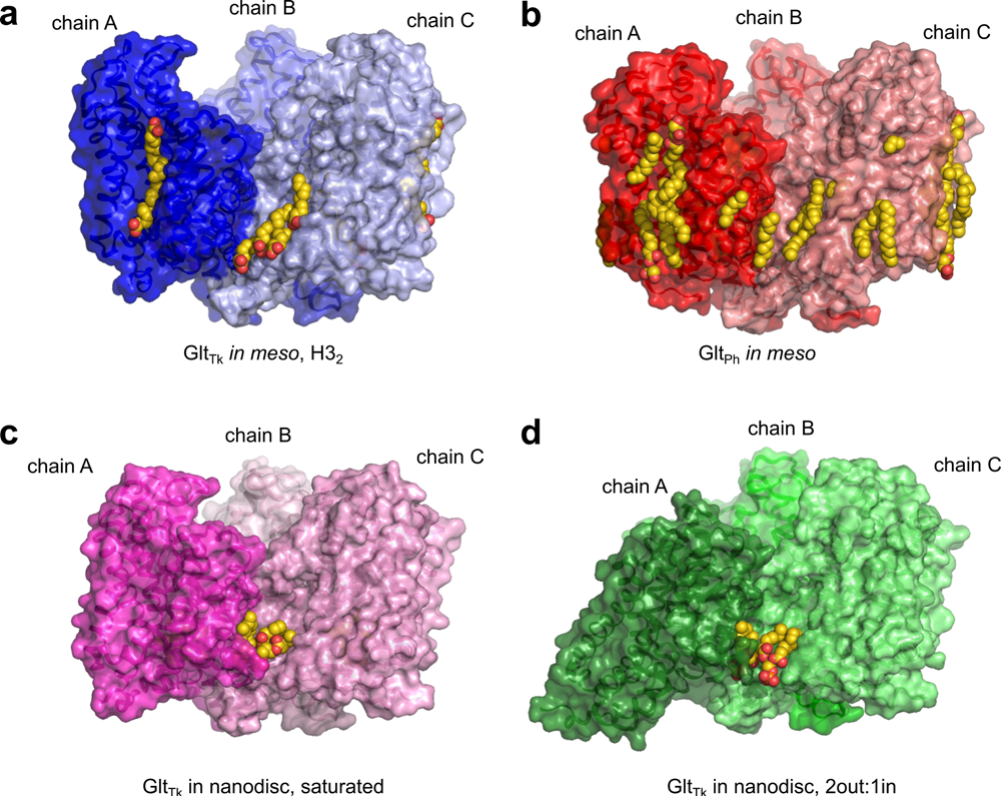
Comparison of lipid positions found in the Glt_Tk_/Glt_Ph_ structures. (a) Glt_Tk_ in *H*3_2_ space groups (this work); (b) structure of Glt_Ph_ obtained *in meso* (7AHK); and (c and d) structures of Glt_Tk_ obtained with cryo-EM (saturated conformation, 6XWQ, and 2out:1in conformation, 6XWP, with additionally modeled lipids). Lipids are shown as vdW spheres (carbon, yellow; oxygen, red).

Importantly, Glt_Tk_ crystallization using the *in meso* approach allows for an unambiguous assignment of elongated densities at the protein hydrophobic interface as lipid molecules (Figure 3), in contrast with the *in surfo* approach that often cannot distinguish between cocrystallized lipids, detergent, and PEG molecules. Analysis of the *in meso* Glt_Tk_ structures obtained here revealed that the hydrophobic groove outside of the trimer interface (between helices TMS2 and TMS5 and helices TMS3 and TMS4c, Figure 2), is filled with lipid fragments, modeled as MO fragments, similarly to what was observed previously in the Glt_Ph_*in meso* structure.^37^

We also extended our analysis of lipid positions to cryo-EM structures, obtained previously^16^ in nanodiscs. To do that, we have reprocessed the previously obtained data and managed to achieve a 0.1–0.2 Å increase in resolution (Supplementary Figure 4). These improvements allowed us to identify multiple densities that cannot be modeled as a part of either a transporter or a nanodisc, and hence were modeled as lipid molecules due to their presence in nanodisc reconstitution lipid mixture.^16^ These molecules are located in a hydrophobic groove between the transport and scaffold domains (helices TMS5 and TMS4c) of adjacent protein chains in all combinations of outward and inward conformations (Supplementary Figure 4).

Interestingly, the lipid positions in the reported crystal structure (corresponding to a 3out saturated conformation) are similar to the lipid positions in the cryo-EM structures of Glt_Tk_ in all conformations: saturated, 1in:2out, and 2out:1in (Figure 3 and Supplementary Figure 4). This observation that the lipids remain at this particular area in all conformational states supports an idea that the lipids might serve as lubricants to facilitate the elevator-like movements of transport domains in these proteins.^20^ Interestingly, similar positions observed in human EAAT1^38^ transporter were suggested as possible binding sites for allosteric modulators, however the development of drugs targeting these transporters is not straightforward given the structural conservation within the family and their overabundance as any significant interference will cause the large excitotoxicity.^39^

## Conclusion

In this work, we present a blueprint to manufacture a humidity chamber for the Gryphon crystallization robot. It is simple and economical to produce and can be easily built in any university workshop. The design is robust and reliable and does not require any extra investments, apart from a simple home appliance humidifier. The box allows performing LCP/*in meso* setups in a humidity protecting environment, preventing the drop evaporation and hence increasing the reliability and reproducibility of crystallization trials. We tested our setup on the Glt_Tk_ membrane transporter, obtaining *in meso* crystals of it for the first time, in contrast to our previous unsuccessful attempts. Obtained crystals were used to collect diffraction data which allowed to unambiguously assign lipid molecules. *In meso* crystallographic data together with reprocessed cryo-EM data allowed us to identify new lipid molecules in functionally relevant locations between the transport and scaffold Glt_Tk_ domains.

## Data Availability

Protein models and corresponding structure factors of Glt_Tk_ obtained using *in meso* crystallization have been deposited in the RCSB Protein Data Bank with PDB codes 8QB4 and 8QB5 for the *H*3_2_ and *P*6_3_22 space groups, respectively.

## Supporting Information Available

Overall design of the assembled humidity box, images of obtained crystals, crystal packing, pipeline of cryo-EM data reprocessing, and data collection and refinement statistics table (PDF)
The blueprint of a humidity box (ZIP)
