# Supplementary material for: Custom Design of a Humidifier Chamber for InMeso Crystallization
Source: Cryst Growth Des. 2023 Dec 12;24(1):325–30. doi: 10.1021/acs.cgd.3c01034 (PMC10767699; doi:10.1021/acs.cgd.3c01034)
Supplement: Supplementary file 1 — cg3c01034_si_001.pdf [file cg3c01034_si_001.pdf]

Supporting information

Custom Design of a Humidifier Chamber for *In Meso* Crystallization

Egor Marin[1], Kirill Kovalev[2], Therese Poelman[1], Rick Veenstra[1], Valentin Borshchevskiy[3], Albert Guskov[1]

[1]University of Groningen, 9747AG, Groningen, the Netherlands

[2]European Molecular Biology Laboratory, EMBL Hamburg c/o DESY, 22607, Hamburg, Germany

[3]Forschungszentrum Jülich, Jülich, Germany

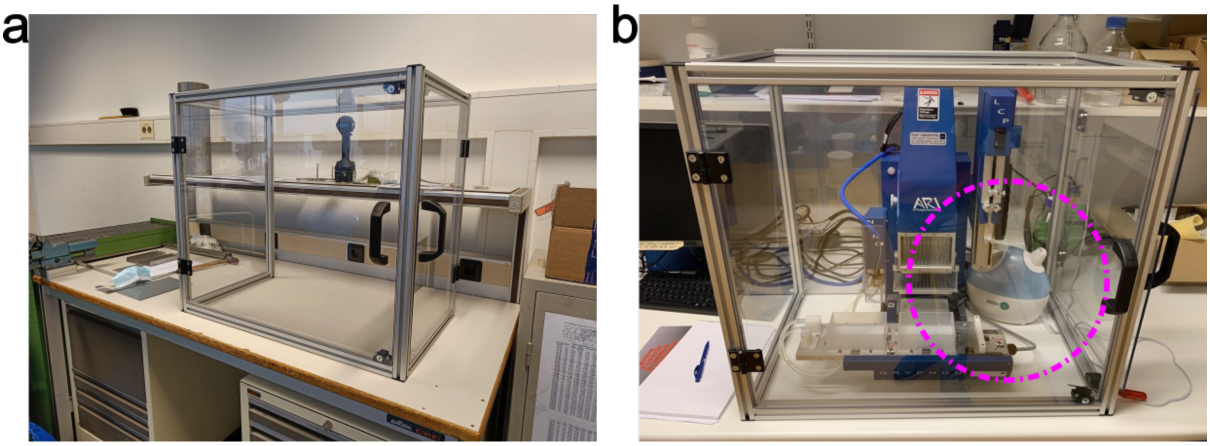

Supplementary Figure 1. Overall design of the assembled humidity box a) without and b) with the Gryphon robot installed. Humidifier is encircled with pink.

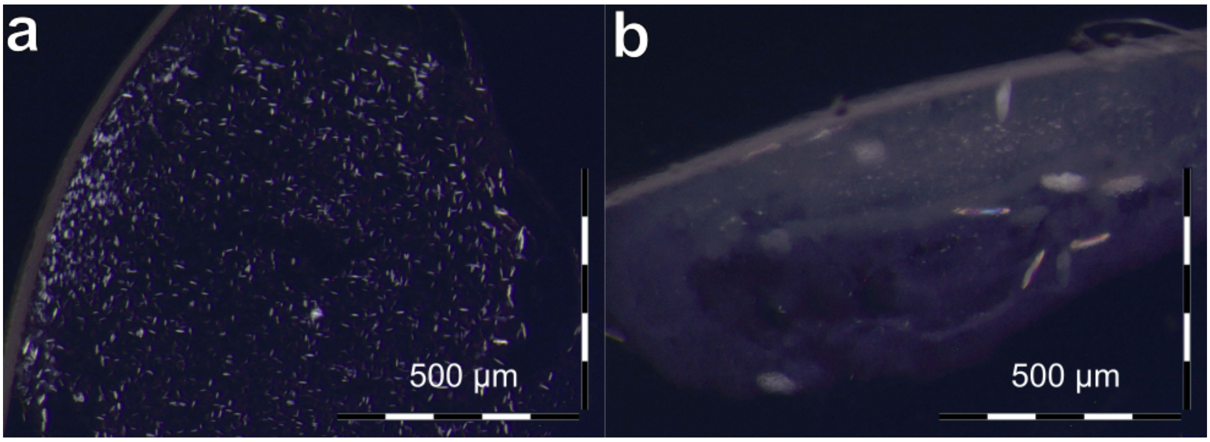

Supplementary Figure 2: Representative pictures of Glt_{Tk} crystals grown *in meso*, varying in sizes from a) 20-50 µm, b) 100 µm.

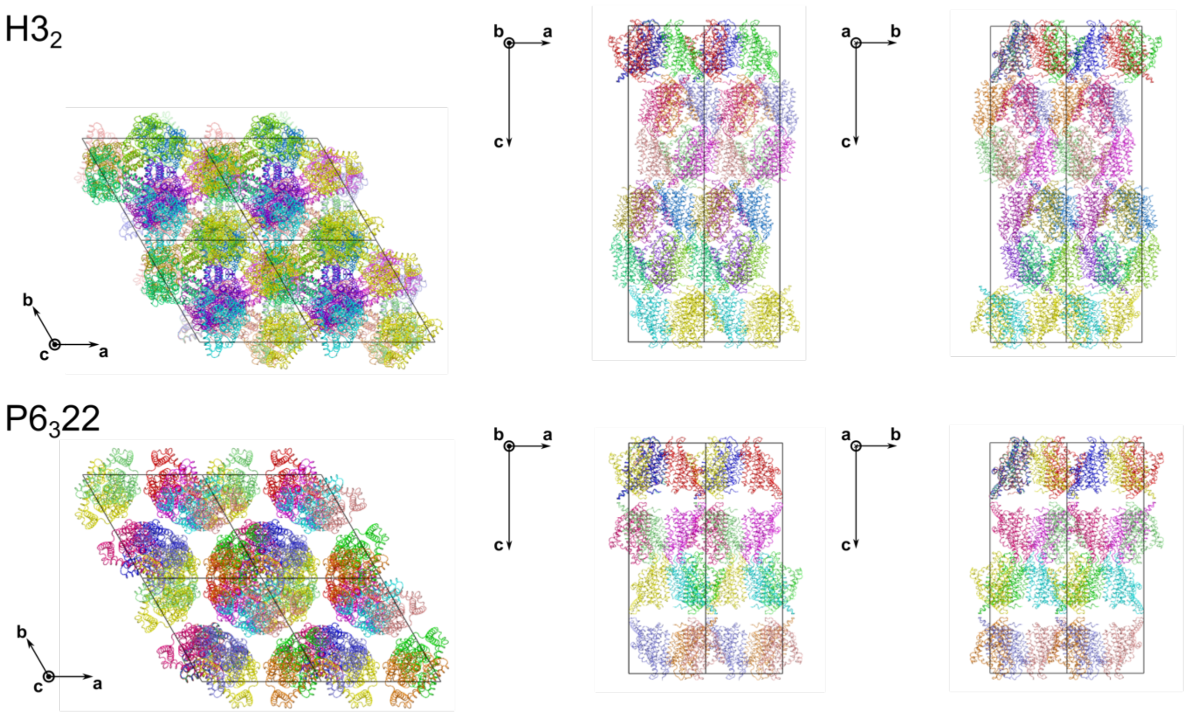

Supplementary Figure 3. Crystal packing of protein molecules for structures obtained in this work, visualized with `supercell` tool in PyMOL (http://www.pymol.org/pymol).

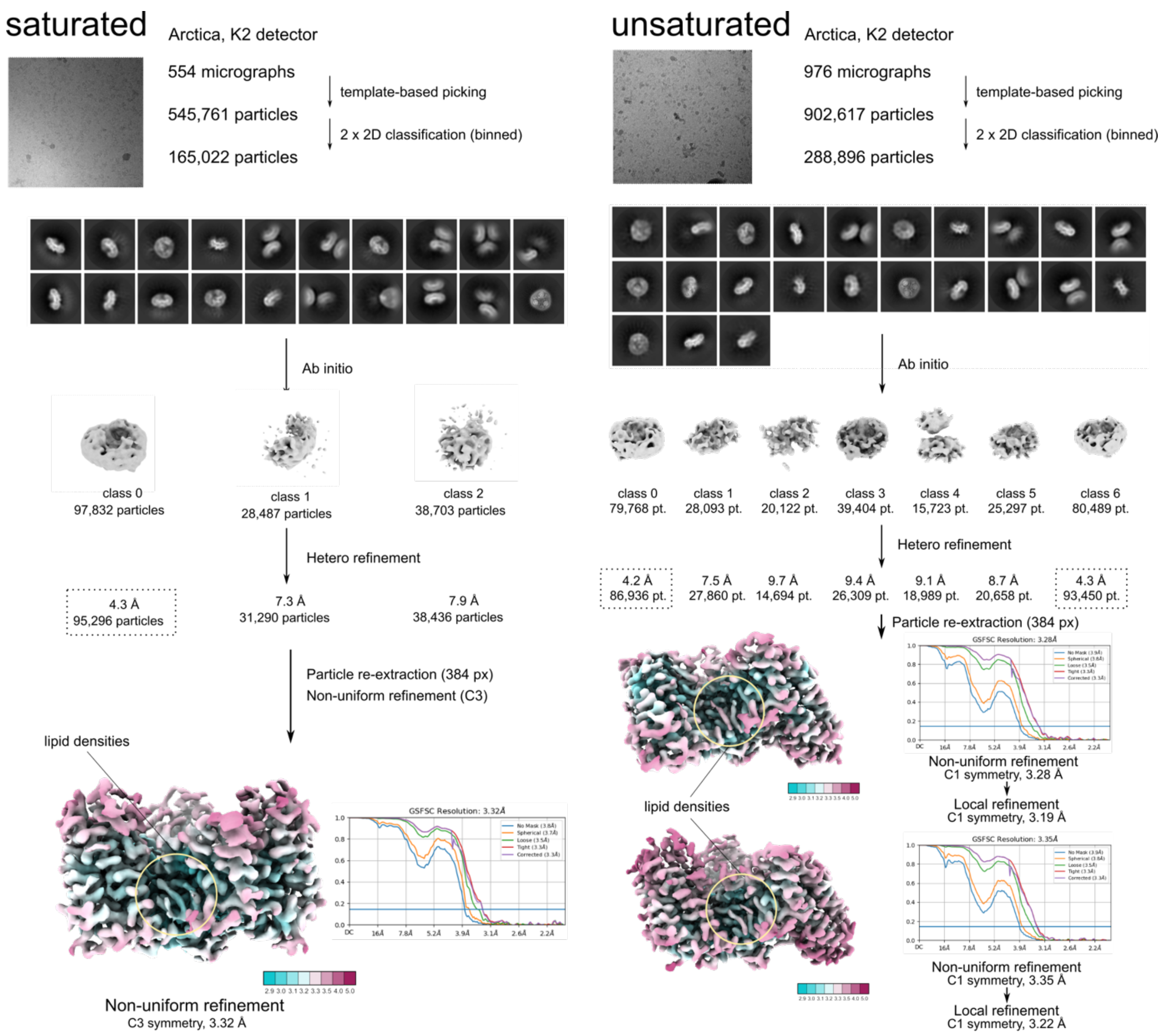

Supplementary Figure 4. Pipeline of cryo-EM data re-processing using cryoSPARC. Densities are colored by local resolution. Nanodisc density is removed via adjusting a map threshold to 0.3-0.4 in ChimeraX (http://www.cgl.ucsf.edu/chimerax/). Lipid densities are circled in light-yellow.

	PDB 8QB4	PDB 8QB5
**Data collection**		
Space group	$H3_2$	$P6_322$
Cell dimensions		
a, *b*, *c* (Å)	92.9 92.9 335.4	94.2 94.2 244.6
α, β, γ (°)	90 90 120	90 90 120
Resolution (Å)	50.0-2.7 (2.77-2.70)	47.1-3.2 (3.3-3.2)
Wilson B-factor	70.2	101.5
R_{meas}, %	34.0 (464.9)	32.2 (396.8)
$I / \sigma I$	8.3 (0.7)	13.0 (1.1)
$CC_{1/2}$, %	99.7 (24.2)	99.8 (58.5)
Completeness (%)	99.2 (100.0)	92.6 (99.9)
Redundancy	19.3 (20.1)	36.9 (39.1)
**Refinement**		
Resolution (Å)	42.9-2.7 (2.8-2.7)	47.1-3.2 (3.5-3.2)
No. reflections	15646 (1538)	10342 (2563)
R_{work} / R_{free}, %	25.4/29.7 (33.2/36.8)	29.2/31.2 (41.1/42.3)
No. atoms		
Protein	3030	2994
Lipids	181	178
Overall	3211	3172
B-factors		
Protein	76.9	102.2
Lipids	82.7	116.4
Overall	77.1	102.5
R.m.s. deviations		
Bond lengths (Å)	0.011	0.004
Bond angles (°)	1.40	0.73

Supplementary Table 1: Data collection and refinement statistics.
